# Supplementary material for: Associations between Bovine Coronavirus and Bovine Respiratory Syncytial Virus Infections and Productivity, Health Status and Occurrence of Antimicrobial Resistance in Swedish Dairy Herds
Source: Antibiotics (Basel). 2021 May 27;10(6):641. doi: 10.3390/antibiotics10060641 (PMC8227817; doi:10.3390/antibiotics10060641)
Supplement: Supplementary file 1 [file antibiotics-10-00641-s001.zip › antibiotics-1213211-supplementary.pdf]

Table 1. Descriptive statistics based on questionnaire data for the 76 herds that fully completed the study.

**Outcome Variables**	**Descriptive Statistics** (unit)					
**Occurrence of Diarrhea and Cough** (% of ratings)						
	**Response rate**	**None**	**A few**	**One fourth**	**Half**	**More than half**
Calf diarrhea	98%	25	57	11	3	4
Young stock diarrhea	96%	84	13	1	1	1
Cow diarrhea	96%	65	30	1	1	3
Calf cough	98%	49	43	7	1	0.3
Young stock cough	97%	71	25	3	1	0
Cow cough	97%	78	17	3	1	1
**Occurrence of other Diseases** (percentiles for all animals in the age category during each 2-month period,)						
	**Response Rate**	**P10**	**P25**	**P50**	**P75**	**P90**
Udder disease	97%	0	1.5	2.9	5.3	8.3
Non-specific fever – cows	94%	0	0	0	0	0.8
Hoof and leg-disorders – cows	97%	0	0	2.1	3.5	5.2
Feed-related disorders – cows	95%	0	0	0	1.1	2.4
Metritis	96%	0	0	0	1.3	2.3
Abortions	95%	0	0	0	0.3	1.5
Umbilical infection – calves	95%	0	0	0	0	0.9
Hoof and leg-disorders – calves	95%	0	0	0	0	2.0
Dull without reason – calves	95%	0	0	0	1.8	4.5
**Antimicrobial Treatments** (percentiles for all animals in the age category during each 2-month period)						
	**Response Rate**	**P10**	**P25**	**P50**	**P75**	**P90**
Lactating cows	97%	0	0	2.1	3.9	7.1
Dry cows	92%	0	0	6.2	17.9	32.4
Young stock	95%	0	0	0	0	0
Calves	96%	0	0	0	3.1	5.7

Table 2. The proportion of resistance, ranges tested, cut-off values, the Minimum Inhibitory Concentrations MIC50 and MIC90 from antimicrobial susceptibility testing of *Escherichia coli* from feces of dairy calves to 13 antimicrobials. 1331 calves from 76 herds.

**Antimicrobial**	**Range Tested** (mg/L)	**Cut-off Value**[1] (mg/L)	**Resistance** (%)	**95% Confidence Interval**	**MIC$_{50}$**[2] (µg/mL)	**MIC$_{90}$**[3] (µg/mL)
Ampicillin	1 – 128	> 8	19.3	17.3; 21.5	2	> 128
Cefotaxime	0.06 – 2	> 0.25	1.1	0.7; 1.9	≤0.06	0.12
Ceftazidime	0.25 – 4	> 0.5	1.5	1.0; 2.3	≤0.25	≤0.25
Chloramphenicol	4 – 32	> 16	3.4	2.5; 4.5	4	8
Ciprofloxacin	0.016 – 1	> 0.06	5.2	4.1; 6.5	0.03	0.06
Colistin	0.5 – 4	> 16	3.4	2.5; 4.5	1	2
Florfenicol	4 – 32	> 16	0.2	0.0; 0.6	8	8
Gentamicin	0.5 – 16	> 2	0.1	0.0; 0.4	≤0.5	≤0.5
Nalidixic acid	2 – 64	> 16	6.5	5.3; 8.0	≤2	4
Streptomycin	4 – 64	> 16	33.5	31.0; 36.1	4	>64
Sulfamethoxazole	8 – 1024	> 64	26.3	24.0; 28.8	16	> 1024
Tetracycline	1 – 128	> 8	18.3	16.3; 20.4	≤1	64
Trimethoprim	0.12 – 16	> 2	6.0	4.9; 7.4	0.25	0.5

[1]Cut-off value according to the epidemiological cut-off values established by EUCAST; [2]MIC50 and [3]MIC90 = MIC that inhibit 50 and 90% of the isolates, respectively.
